# Radial Head Resection versus Arthroplasty in Unrepairable Comminuted Fractures Mason Type III and Type IV: A Systematic Review

**DOI:** 10.1155/2018/4020625

**Published:** 2018-07-16

**Authors:** Francesco Catellani, Francesca De Caro, Carlo F. De Biase, Vincenzo R. Perrino, Luca Usai, Vito Triolo, Giovanni Ziveri, Gennaro Fiorentino

**Affiliations:** Department of Orthopaedics and Traumatology, Istituto Clinico Humanitas Gavazzeni, Bergamo 24125, Italy

## Abstract

Unrepairable comminuted fractures of the radial head Mason type III or type IV have poor outcomes when treated by open reduction and internal fixation. Radial head resection has been proposed as good option for surgical treatment, while in the last decades, the development of technology and design in radial head prosthesis has increased efficacy in prosthetic replacement. The present review was conducted to determine the best surgical treatment for comminuted radial head when ORIF is not possible. Better outcomes are reported for radial head arthroplasty in terms of elbow stability, range of motion, pain, and fewer complications compared to radial head excision. Nevertheless, radial head resection still can be considered an option of treatment in isolated radial head fractures with no associated ligament injuries lesion of ligaments or in case of older patients with low demanding function.

## 1. Introduction

Surgical treatment for comminuted and unrepairable fractures of the radial head may be challenging. These types of fractures are often associated with multiple ligamentous injuries amounting to elbow instability. Radial head resection has been proposed as good option for surgical treatment, while in the last decades, the development of technology and design in radial head prosthesis has increased efficacy in prosthetic replacement.

The radial head is a secondary valgus stabilizer of the joint and it is involved in transmission of axial force load through the elbow during flexion [1]. It is also a varus and external rotatory constrainer [2]. Comminuted radial head fractures Mason type III and type IV are commonly associated with other injures of the elbow as capitellum and coronoid fractures and/or ligaments disruption, both medial and lateral ligaments and interosseus membrane [3–6]. Primary goal in surgical treatment is to restore elbow stability in order to preserve the complex physiologic elbow kinematics. In this respect, medial collateral ligament is the primary constrainer in valgus stress. Radial head contributes secondarily to valgus stability [1, 7] and its preservation is mandatory in case of fractures that involve soft tissue and ligaments to avoid chronic instability. Many authors have described serious complications in case of resection of the radial head such as proximal migration of radius and longitudinal instability, humeroulnar osteoarthritis [2, 7–9], decrease in grip strength, cubitus valgus, and ulnar neuropathy [10, 11]. Therefore, radial head arthroplasty has obtained a large consensus in orthopaedic surgeons as primary option of treatment in fractures Mason types III and IV. It allows an anatomical reconstruction and it maintains stability and physiologic kinematics of the elbow if associated with ligament reconstruction. However, oversizing or overstuffing of radial head prosthesis, malpositioning, and aseptic mobilization may lead to a high rate of complications and failure of this surgical procedure. Recent reviews of literature [10, 12] on elbow arthroplasties have reported satisfactory results in radial head replacement studies due to improvement of biomaterials and operative techniques.

The purpose of this review was to investigate the current literature on surgical treatment of unrepairable comminuted radial head fractures Mason type III or type IV to assess results and indications for radial head replacement or resection.

## 2. Materials and Methods

**Figure 1:**
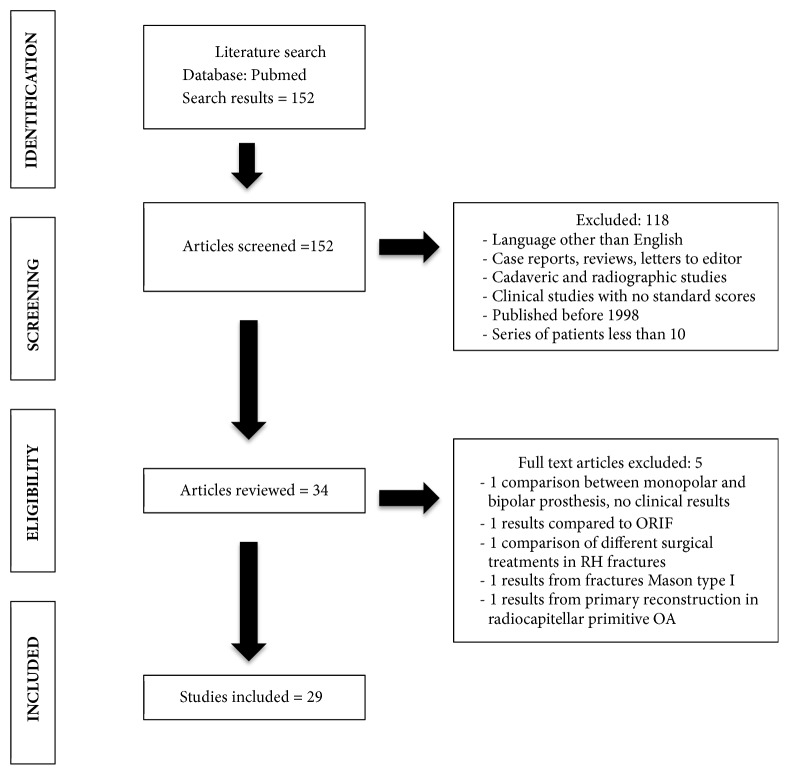


We searched in PubMed electronic database the words (radial head fractures) AND ((artrhoplasty) OR (prosthesis)) AND ((resection) OR (excision)). The guidelines for preferred reporting items for systematic reviews and meta-analysis (PRISMA) were used (Figure 1). We selected articles of the last 20 years, from 1998 to December 2017. We created an Excel database for collecting data extracted from articles in English language, selecting papers with series of 10 or more patients. Exclusion criteria were articles written in other languages, case reports or reviews, cadaveric or instrumentals studies, clinical studies with no standard questionnaires or scores, and studies in which posttraumatic outcomes were not separated from primary reconstruction of the radial head.

We extracted relevant data from the selected articles: type of study, number of patients, age, follow-up, type of surgery performed, clinical results (ROM, DASH score, MEPS score, and VAS), and radiographic results.

## 3. Results

The database search identified 152 potentially relevant articles. Abstracts have been analyzed following inclusion and exclusion criteria and a total of 29 papers were selected for the present review. Most of retrospective studies on metal radial head prosthesis have been published in the last ten years in comparison to a lack of studies for radial head excision in the last two decades. Moreover, few articles on comparison of the two surgical techniques have been found. Because of heterogeneity in level of evidence, surgical technique, type of implants, and rehabilitation protocol, we did not perform statistical data analysis. Articles selected are reported in Table 1.

## 4. Discussion

From our review of literature clinical results for radial head replacement are reported in Table 2. Most of retrospective studies involve modular monopolar or bipolar prosthesis implanted for irreparable Mason type III or type IV fractures. For most of authors, mid term follow-up has shown satisfactory results in range of motion recovery (average flexion-extension arc of motion: 116°). Good results in DASH scores (from 7 to 24) and MEPS scores (from 79 to 100) and low VAS pain evaluation scale (from 0 to 2.2) are reported [13–32]. A certain loss of grip strength compared to contralateral side is often described (average loss of strength: 10% respect to the contralateral side). Authors highlight the importance of ligament reconstruction in case of associated injuries. Intraoperative assessment of stability and acute repair of torn ligaments is mandatory for a successful procedure.

Most common radiological modifications include osteoarthritic changes of ulnohumeral joint, capitellum wear for oversizing of radial head prosthesis, periarticular heterotopic ossifications, and radiolucency lines around the stem. Some modifications in radiographic appearance seem to not correlate directly with clinical symptoms: bone resorption around the prosthesis does not correlate with loosening of the prosthesis and does not affect clinical scores. Marsh [21] reports favorable clinical outcomes from short to long follow-up despite a high evidence of radiolucency around the stem and arthritis in his series. Gauci [20] has found no association between neck bone resorption and postoperative symptoms.

Complications (Table 3) described in radial head replacement are in common in almost all the papers: aseptic mobilization of the stem, overstuffing, erosion of the capitellum, osteoarthritis, and heterotopic ossification clinically arising with lateral elbow pain or loss of motion, and posterior subluxation for undersizing. Rare temporary ulnar and radial nerve sensory neuropathies are reported. Though, few papers seem to discourage radial head arthroplasty. Moro [31] reports mild to moderate impairment of ROM and pain for both elbow and wrist in patients treated with a metal radial head implant. Laumonerie [16] describes unsatisfactory result from bipolar radial head prosthesis because of malposition in varus and valgus and oversizing leading to a high rate of reintervention during the three first months after implantation. Flinkkila [23] reports poor results from press fit radial head prosthesis due to a high rate of loosening. Difficulties on technique of implantation are described by Ashwood [30] for mono-block prosthesis.

Retrospective studies on radial head resection have a longer follow-up and clinical and radiological results are reported in Table 4 [33–42]. Clinical and radiological complications at long-term follow-up are reported (Table 5). Clinical results show good outcomes in Mayo Elbow Performance Scores (MEPS, from 79 to 100) and Disabilities for Arm Shoulder and Harm scores (DASH, from 4 to 15), a satisfactory recovery of elbow range of motion (average flexion-extension arc of motion: 120°) and low scores in VAS scale (from 0 to 4.6). However common complications of this surgical procedure involve ulnohumeral joint due to an higher load compression force that leads to degenerative changes and progressive worsening of cubitus valgus associated to ulnar nerve neuropathy and UCL elongation leading to chronic elbow instability [3, 4]. Moreover, proximal migration of radius is often assessed (80% of papers), complications that involve DRUJ impairment leading to wrist pain hand strength reduction and distal radio-ulnar arthritis. Preoperative or intraoperative setting of elbow stability and correction of ligaments injuries is mandatory to avoid early complications. Despite of complications, many authors approve the surgical technique due to good outcomes in mid to large term. Yalcinkaya [36] found no significant correlation between radiological degenerative modifications in elbow and outcomes of clinical scores in patients treated by radial head resection. Antuna [38] reports good clinical results in a large series of patients less than forty years old treated by radial head excision after a mean follow-up of 25 years. Herbertsson [39] reports worst outcomes in excision for Mason type IV fractures although delayed radial arthroplasty is suggested for pain relief and preservation of range of motion in case of failure of other treatments.

Finally, few papers compare radial head resection and radial head arthroplasty [34, 35] where authors recommend resection as primary option of treatment because of a lack of statistical clinical differences between the two surgical procedures, in case of isolated radial head fractures not associated to ligaments injuries. Nestorson [33] did not found better outcomes by using a press fit radial head prosthesis in Mason type IV fractures and he reports similar functional results after radial head resection despite more osteoarthritic changes. Lopiz [34] suggests resection as a good option of treatment when ORIF is not possible, reporting a higher rate of complications in the group of patient treated by radial head arthroplasty.

## 5. Conclusion

From our review of literature almost all the retrospective studies on radial head arthroplasty report convincing results in terms of elbow stability, range of motion, and pain. Nevertheless, papers on radial head resection report good clinical outcomes in isolated radial head resection with no associated ligament injuries. Few papers compare the two techniques with no substantial differences in terms of clinical outcomes at medium and long follow-up.

## Figures and Tables

**Table 1:** Studies selected. Number and age of patients, type of treatment, and follow-up.

Author	Type of study and year of publication	N. of patients	Age	Type of treatment	Follow up
Carità E	Retrospective 2017	28 (Mason type III – IV)	49 yo (18-71)	Cementless monopolar prostheses (Acumed – Tornier)	49 months (6-118)

Laflamme M	Retrospective 2017	46 (21 Mason III; 36 Mason IV)	Porous stem: 52.8 yo Smooth stem: 45.6	Modular monopolar head – uncemented loose fitting stem (Evolve, Wright) Modular monopolar head - porous press-fit stem (ExploR, Biomet)	6,3 years (1,2-15,1)

Tarallo L	Retrospective 2017	31 Mason III	-	31Radial head replacement (Anatomic RHA, Acumed)	30 months (12 months to 7 years)

Nestorson J	Restrospective 2017	32 Mason IV	50 yo (29-70)	18 pts radial head arthroplasty 14 radial head resection	58 months (RHA) 108 months (RH resection)

Laumonerie P	Retrospective 2017	77 (65 Mason type III, 2 Mason type II; 10 radial neck fractures)	52 yo (20-82)	Guepar radial head prosthesis (SBi/Stryker) Evolutive (Aston Medical) rHead Recon (SBi/Stryker) rHead STANDARD (SBi/Stryker)	74 months (24 to 141)

Lopiz	Retrospective 2016	25 Mason III	Excixion 53 yo Arthroplasty 54.4yo	11 patients radial head resection 14 Radial head prosthesis	Excision 60.3 months Arthroplasty 42 months

Van Hoecke	Retrospective 2016	21 Mason III	53,2 yo	Judet bipolar head prosthesis	113 months

Heijink	Retrospective 2016	25 Mason type III	55 yo	Cemented bipolar radial head artrhoplasty (Tornier)	50 months

Kodde	Retrospective 2016	27	48 yrs (24-63)	Press fit bipolar radial head arthroplasty	48 months (28-73)

Marsh JP	Retrospective 2016	55	61 yrs	Modular smooth-stemmed radial head implant (Evolve, Wright)	8.2 yrs

Gauci MO	Retrospective 2016	65 (10 ORIF revision 42 Mason III 12 post traumatic radiohumareal sequelae, 1 swanson prosthesis revision)	52 yrs (22-85)	Modular Pyrocarbon (MoPyc) radial head prosthesis (Tornier)	42 months (24-108)

Solarino G.	Retrospective 2015	30 (12 Mason II; 18 Mason III)	71 yo (65-80)	Radial head resection	40 months (24-72)

Allavena C	Retrospective 2014	22 (16 fractures Mason type III; 6 fractures of the radial neck)	44 yrs (22-65)	Modular bipolar radial head prostehesis (Guepar,De Puy)	50 months

Yalcinkaya M	Retrospective 2013	14 fractures Mason type III	38 yrs (20-67)	Radial head resection	14,7 yrs (9-26)

Flinkkila T.	Retrospective 2012	42 (34 Mason type III; 8 type II)	56 yrs (23-85)	Metallic radial head artrhoplasty	50 months (12-107)

Sarris IK	Retrospective 2012	5 Mason type III; 15 type IV; 10 complex elbow injuries; 2 malunion	54 yrs (32-68)	MoPyc pyrocarbon prosthesis (Bioprofile, Tornier)	27 months (21-46)

Ricon F	Retrospective 2012	28 Mason III	54 yrs	Pyrocarbon radial head prosthesis (Bioprofile Lab.)	32 months (12-62)

Muhm M	Retrospective 2011	25 radial head fractures type III and type IV	-	Uncemented modular metallic prosthesis (Evolve)	Short term 1,6yrs Mid term 5,1 yrs

Iftimie	Retrospective 2011	22 (16 Mason type III; 6 type IV)	54 yrs (28-81)	Resection head arthroplasty	16,9 yrs (10-24)

Celli A	Retrospective 2010	16 patients (9Mason type III 7 Mason type IV)	46.1 yrs (27-74)	Bipolar Judet radial head arthroplasty (Tornier)	41,7 months (12,3 – 86,3)

Antuna SA	Retrospective 2010	26 patients (6 type III 20 type IV)	29 yrs (15-39)	Radial Head Resection	24,9 yrs (15-34)

Dotzis A	Retrospective 2006	14 patients (6 Mason type III; 8 type IV)	44.8 years (18 – 85)	Judet prosthesis (Tornier)	5.3 years (1-12 yrs)

Ashwood N	Retrospective 2004	16 Mason type III	45 yrs (21- 72)	Metallic monoblock radial head prosthesis (Wright Med Tec.)	2.8 years (1.2-4.3)

Herbertsson P.	Retrospective 2004	61 patients 39 Mason type II 10 Mason III 12 Mason IV	44 yrs (9-69)	Radial head resection Primary RHE=39 Delayed RHE=18	18 years (11-33)

Moro JK	Retrospective 2001	25 (10 Mason type III;15 Mason type IV)	54 yrs	Metal Radial head arthroplasty	39 months

Sanchez Sotelo J.	Retrospective 2000	10 Mason type III	39 yrs (26-57)	Radial head resection	4.62 years (24-86 months)

Ikeda M	Retrospective 2000	11 Mason type III	40 yrs (25-70)	Radial head resection	11 years (3-18)

Smets A	Retrospective 2000	13 Mason type III	-	Floating radial head prosthesis	25.2 months

Jansen RP	Retrospective 1998	18 Mason III	-	Radial head resection	16 to 30 years

**Table 2:** Mean clinical results for radial head arthroplasty.

Author	Type of prosthesis	ROM	VAS	DASH	Meps/Mepi	Other clinical evaluations
Carità E	Cementless monopolar prostheses (Acumed – Tornier)	Flexion- extension arc 107° pronosupination 159°	1.8	14,2	Meps 89	Patient Rated Wrist Evaluation score (PRWE) = 29

Laflamme	Modular monopolar head – uncemented loose fitting stem (Evolve, Wright) Modular monopolar head - porous press-fit stem (ExploR, Biomet)	Mean elbow flexion difference compared with the normal side: 4°; extension 14 pronation 8° supination 15°	1.11	7.7	Mepi 96.5	Grip strength compared with the normal side (Jamar dynamometer kg/force): 1.0 (-24-13)

Tarallo	Anatomic RHA, Acumed)	Flexion-extension arc 112° (95°-112° Pronosupination 134°	-	-	Meps: 24 excellent (77%) 3 good (10%) 4 fair (13%)	-

Laumonerie	Guepar radial head prosthesis (SBi/Stryker) Evolutive (Aston Medical) rHead Recon (SBi/Stryker) rHead STANDARD (SBi/Stryker)	*Acute treatment* Flexion 132° Extension -12.9° Supination 67.8° Pronation 76°	-	13.1	Meps 91.5	Force compared to contralateral side: flexion 87.2 extension 93.6

Nestorson	Radial head arthoplasty	Flexion-extension arc 130° (95°-155°) Forearm rotation 30° (10°-85°)	-	13	Meps: 85	-

Lopiz	Radial head arthoplasty	Flexion-extension arc 85.5°	-	24.8	Meps: 6 Excellent 3 good 2 fair 2 poor	-

Van Hoecke	Judet bipolar head prosthesis	Flexion 121.8° Extension 24,8° Pronation: 62.4° Supination 58.8°	-	23.1	Mepi 88,6	-

Heijink	Cemented bipolar radial head artrhoplasty (Tornier)	Flexion-extension arc 129° Forearm rotation 131°	Pain: 13 absent 7 mild 3 moderate 1 severe	-	Meps 13 Excellent 7 good 3 fair 1 poor	-

Kodde	Press fit bipolar radial head arthroplasty (Tornier)	Flexion-extension arc 126° Forearm rotation 138°	Pain: 17 absent 3 mild 7 moderate	-	Meps 17 Excellent 2 good 7 fair 1 poor	-

Marsh	Modular smooth-stemmed radial head implant (Evolve, Wright)	Flexion-extension arc 126°+/- 21° Pronation 79° Supination 67°	-	14	Mepi 91+/- 13 points	Patient-Rated Elbow Evaluation (PREE): 14 Mean grip strength: 97% of that of the unaffected limb

Gauci	Modular Pyrocarbon (MoPyc) radial head prosthesis (Tornier)	Flexion 136° Extension -9° Pronation 71° Supination 76°	1		Meps 96	-

Allavena	Modular bipolar radial head prostehesis (De Puy)	Flexion-extension arc 100° Rotation arc 143°	-	21	Meps 79	Mean wrist strength 86% compared to contralateral side Mean elbow strength 67% compared to contralateral side

Flinkkila T.	Metallic radial head artrhoplasty	Flexion-extension arc 117° extension deficit 20°	-	23	Meps 86	-

Sarris IK	MoPyc pyrocarbon prosthesis (Bioprofile, Tornier)	Flexion-extension arc 130° Pronation 74° Supination 72°	-	-	Meps excellent 80% good 17% fair 3%	Mean grip strength 96% compared to contralateral side

Ricon F	Pyrocarbon radial head prosthesis (Bioprofile Lab.)	Flexion-extension arc 105° Pronation 85° Supination 80°	-	-	92	Mean grip strength reduced of 10% on the injured side

Muhm M	Uncemented modular metallic prosthesis (Evolve)	mid-term (15 patients) flexion 127.3 extension 15.7 pronation 74.3 supination 71.7	-	24,9	-	Broberg and Morrey scoring system 85,2

Celli A	Bipolar Judet radial head arthroplasty (Tornier)	Flexion-extension arc 117° Pronosupination 120°	1.38 at rest 2.25 at work	11.4	Meps 89.4	-

Dotzis A	Judet prosthesis (Tornier)	Flexion-extension arc 14°-140° pronation 87.5° supination 84°	-	23.9	Excellent 6 Good 4 Fair 1 Poor 1	Mean grip strength 90% compared to contralateral side

Ashwood N	Metallic monoblock radial head prosthesis (Wright)	Loss of flexion 10° Loss of pronation 12° Loss of supination 12°	17 (0-100 vas scale)	-	87	Mean grip strength reduced of 12% on the injured side

Moro JK	Metal Radial head arthroplasty	Flexion 140° Extension -8° Pronation 78° Supination 68°	-	17	Mepi 80 Excellent, good 17 Poor 3 Fair 5	SF-36 score: physical component 47; mental component 49 Mean PRWE score: 17 Mean WOS score: 60

Smets A	Floating radial head prosthesis		-	-	Mepi Excellent 7 Good 3 Fair 1 Poor 2	-

**Table 3:** Complications in radial head replacement.

Author	N. of patients	Complications
Carità E	28	1 osteolysis and stem mobilization 1 overstuffing (erosion of the capitellum) 2 periprosthetic calcification(asymptomatic) 6 resorption of the neck of the radius (asymptomatic) 3 removal of the implant (1 mobilization; 3 painful elbow)

Laflamme M	46	22 osteolysis >2mm (48%) 4 Overstuffing 1 degenerative changes (Broberg and Morrey grade III) 5 heterotopic ossification Brooker grade II, 1 grade IV

Tarallo L	31	8 heterotopic ossification (26%) 2 radiolucent lines (asymptomatic)

Nestorson J	18	4 surgical revision (3 aseptic loosening, 1 proximal radio-ulnar synostosis, 1 CPRS) 5 late osteoarthritis

Laumonerie P	54 acute injuries 23 delayed surgery	8 painful loosening 4 radiohumeral conflict 3 radiocapitellar instability 5 ulnar nerve palsy 4 CPRS 30 reoperations (38.9%) (19 implants removed; 11 retention of the implant)

Lopiz	14	3 elbow stiffness 2 oversizing 1 periprosthetic fracture 2 neuropathies (ulnar and radial) 4 elbow arthritis grade I, 9 cases grade II, 1 case grade III (Broberg and Morrey classification) 11 periarticular ossification (asymptomatic) 5 bone lucencies (asymptomatic)

Van Hoecke	21	14 capitellar erosion 10 ulnohumeral arthritis 1 radiolucent lines 1 overlenghtening 1 ulnar plus 1 prosthesis removed

Heijink	25	3 radiolucency lines (asymptomatic) 5 periarticular ossification (asymptomatic) 7 osteolysis of proximal radius (asymptomatic) 4 erosion of the capitellum 13 ulnohumeral arthritis 2 radial nerve neuropraxia 1 luxation (dissociation of the prostheses) – removed 2 subluxation

Kodde IF	27	3 revisions for chronic instability 5 revision for ulnar nerve dysfunction, elbow stiffness, symptomatic arthritis 23 radial neck osteolysis 13 ulnohumeral degeneration 7 erosion of the capitellum 5 heterotopic ossification (asymptomatic) 1 posterior subluxation 2 persistent pain for medial and lateral epicondylitis

Marsh JP	55	25 periprosthetic lucency 21 ulnohumeral arthritis 20 heterotopic ossification 12 capitellar osteopenia 1 abnormal radiocapitellar alignment

Gauci MO	65	48 (92%) cortical resorption around prosthesis neck 9 capitellum wear 1 radio-ulnar synostosis

Allavena C	22	6 early posterior subluxation 5 sensory ulnar nerve dysfunction 2 CPRS type I 3 lateral elbow pain 1 symptomatic loosening 8 osteolysis 1 advanced osteoarthritis 6 capitellar erosions 4 anterior ossifications

Flinkkila T.	42	1 infection 1 radial nerve palsy 21 osteoarthritis (3 severe) 14 capitellar erosion 9 prostheses removed (6 painful, 2 loosed)

Sarris IK	32	2 stem-neck dissociation 1 stiffness 2 periprosthetic lucencies (asymptomatic) 7 heterotopic ossification (asymptomatic) 4 radiographic sign of stress shielding (asymptomatic)

Ricon F	28	2 posterior subluxation (overstuffing) 11 radial neck resorption 5 ossification in collateral ligament 1 mild periprosthetic ossification

Muhm M	Mid term 15	12 periprosthetic radiolucency 12 (70%) heterotopic ossification 12 (70%) osteoarthritis

Iftimie	22	24 degenerative changes

Celli A	16	2 heterotopic ossification 2 proximal radio-ulnar synostosis 2 capitellar erosion (overstuffing) 1 proximal bone resorption

Dotzis A	14	1 CPRS and stiffness 1 periprosthetic lucency 7 heterotopic ossification (asymptomatic)

Ashwood N	16	1 CPRS 3 ulnar neuropathies 2 superficial wound infections

Moro JK	25	17 bone radiolucency (asymptomatic) 1 CPRS 1 ulnar neuropathy 1 PIN palsy 1 elbow stiffness 1 wound infection

Smets A	13	3 wrist pain 1 implant removed for stiffness

**Table 4:** Mean clinical results for radial head resection.

Author	ROM	VAS	DASH	Meps/Mepi	Other clinical evaluations
Nestorson J	Flexion-extension arc 127,5° (105°-150°)	-	12	Meps: 100	-

Lopiz	Flexion-extension arc 105.2°	-	13.5	Meps 6 excellent 3 good 2 fair 0 poor	-

Solarino G.	flexion 124° extension -11° pronation 78° supination 82°	Pain Absent 14 Mild 8 Moderate 8	13	Meps 79	-

Yalcinkaya M	Flexion-extension arc 127° Pronation 83,2° Supination 84,6°	4,6	6,6	Meps 88,6	-

Iftimie	flexion 135° extension -5° pronation 83° supination 79°	0.48	4,89	-	Grip strength 88% compared to the contralateral side

Antuna SA	flexion 84° extension -9° pronation 84° supination 85°	9	6	95	Grip strength loss 16% compared to contralateral side

Herbertsson P.	*Primary RHA* flexion 140° extension -7° supination 77° pronation 85°	-	-	-	Steinberg system for clinical outcomes: 25 good; 26 fair; 10 poor 28 no symptoms, 27 occasional pain; 6 daily pain

Sanchez Sotelo J.	Flexion-extension 7.5- 140 Pronation 85.5° Supination 83.5°	0	0.66 to 15.9	-	Grip strength loss 15% compared to contralateral side Broberg and Morrey performance index: excellent 5; good 5; poor 1

Ikeda M	flexion 132° extension -14° supination 82° pronation 80°	-	-	-	Grip strength loss 17% compared to contralateral side

Jansen RP	-	-	-	Mepi Excellent 17 Good 3 Fair 1 Poor 2	-

**Table 5:** Complications in radial head resection.

**Author**	**N. Of patients**	**Complications**
Nestorson J	14	2 surgical revision (stiffness) 1 ulnar nerve dysfunction 1 radial nerve dysfunction 13 late osteoarthritis

Lopiz	11	Average radial shortening 2.3mm 1 elbow stiffness 1 valgus instability All patients: elbow arthritis grade I 2 heterotopic ossification (asymptomatic)

Solarino G.	30	Arthritic changes: 4 mild; 3 moderate 5 heterotopic ossification 5 ulnar minus (mean value 3.5) and DRUJ instability

Yalcinkaya M	14	8 degenerative changes in elbow 4 degenerative changes in wrist 3 heterotopic ossification 8 proximal migration of radius Mean ulnar variance: 1.7mm Mean carrying angle 11.2°

Iftimie	22	24 Degenerative changes (Broberg and Morrey 1 patient grade 3; 13 grade 2; 10 grade 1

Antuna SA	26	2 postero-lateral instability 2 valgus laxity 1 DRUJ instability Osteoarthritic changes (17 mild; 9 moderate) 8 heterotopic ossification (asymptomatic) Average radial shortening 3.1mm

Herbertsson P.	61	16 ulnar plus >2 mm Degenerative changes: 42 cysts; 40 irregular subchondral bone; 43 osteophytes

Sanchez Sotelo J.	10	4 heterotopic ossification 8 degenerative arthritis mean proximal radius migration: 1.6mm mean carrying angle decrease: 5.4°

Ikeda M	11	Mean ulnar variance +1.6mm Mean increase in carrying angle 8° Mild to severe degenerative arthritis in all patients

Jansen RP	18	ROM limitations 11 Degenerative changes 7 increase of cubitus valgus, 7 periarticular ossification, 7 osteoporosis of capitellum, 12 proximal radius migration (from 1 to 5 mm)
